# Qigong and a Tale of Two Back Complaints

**DOI:** 10.3390/medicines5030060

**Published:** 2018-06-21

**Authors:** Joseph Baumgarden, Penelope Klein, George Picard

**Affiliations:** 1 Physical Therapy Department, D’Youville College, Buffalo, NY 14201, USA; kleinqpj@gmail.com; 2 Village of Healing and Wellness, St. Catharines, ON L2R 3L2, Canada; george@georgepicard.com

**Keywords:** Qigong, back pain, case study

## Abstract

**Background:** Chronic back pain is one of the leading causes of disability and decreased quality of life for people in both their personal and professional lives. In addition to modern medical intervention, many individuals seek relief through complementary and alternative therapies. **Design:** This study was designed as a retrospective descriptive review presented as two case studies. **Methods:** Data were collected through face-to-face interviews with two volunteer subjects of convenience who each had a medical diagnosis of chronic back pain. **Intervention:** Both individuals practiced a daily, standardized regimen of 24-Posture Qigong. **Results:** Both individuals experienced clinically significant symptomatic relief and functional benefit from their practice of qigong. **Conclusion:** Positive outcomes are theoretically attributed to relief of inflammation systemically and locally, reversal of sensitization and structural restorative reparation. These results provide justification for further prospective, controlled, long-term investigations.

## 1. Background

According to the National Institute of Health, the vast majority (>75%) of adults will experience lower back pain (LBP) over the course of their lives. It is the leading cause of job-related disability and results in over 150 million work days and $16 billion annually in decreased productivity [1,2,3]. In a large survey, more than a quarter of adults reported experiencing daily LBP during the past three months [4]. Results reported in the Global Burden of Disease 2010 study showed that out of 291 conditions studied, LBP ranked highest in years lived with disability. From 1990 to 2010, the number of years lost due to ill-health increased from 58.2 million to 83.0 million. Authors concluded that this was in part due to population growth, and an aging population [5]. Overall, the cost of LBP-related healthcare utilization in the United States is approximately $100 billion a year. It is also the fifth most common reason for physician visits [2,3,6].

In a discussion by Chien, it was highlighted that the mechanical causes of LBP may include sprains and strains, muscle weakness and instability, disc herniation, disc degeneration, arthritis, nerve compression, and stenosis [7]. Conventional medical treatments frequently used to treat LBP include physical therapy, injections, medications such as opioids or muscle relaxants, bracing, and surgery [7]. Many people suffering from LBP are turning to complementary and alternative medicine (CAM) for pain relief as they have found conventional medical treatments to be ineffective for managing their symptoms [8]. In 2015, over 40% of people affected by LBP used some form of CAM, including mind-body therapies such as Tai Chi and Qigong [9]. Estimates based on the 2002–2008 Medical Expenditure Panel survey found that those with spine problems who use CAM reported much higher subjective health states, education, and comorbidity compared with non-CAM users. They also reported $424 lower spine-related costs and $796 lower total health care cost than those who do not use CAM. Differences in spending were related to lower hospital costs among CAM users [9]. In another survey, respondents reported that they chose CAM for their LBP due to it being more effective and having fewer potential side-effects [10].

When comparing CAM practices defined as movement therapies (such as Tai Chi and yoga) to manual therapies (such as chiropractic manipulation and massage), movement therapies were found to be twice as likely to affect emotional health compared to manual therapies [11]. In addition, 80% of people who used movement therapy reported improved physical health (compared to only 69% of those receiving manual therapy). While both forms of CAM therapies can have positive effects on people with arthritis, it appears that CAM therapies that involve active participation in the process can benefit the user more than passive therapies [11].

One of the CAM practices utilized by people searching for relief of LBP symptoms or conditions is qigong. According to the National Qigong Association, Qigong can be described as “a mind-body-spirit practice that improves one’s mental and physical health by integrating posture, movement, breathing technique, self-massage, sound, and focused intent. There are likely thousands of qigong styles, schools, traditions, forms, and lineages, each with practical applications and different theories about *Qi* (“subtle breath” or “vital energy”) and *Gong* (“skill cultivated through steady practice”)” [12].

Written records of the practice of Qigong can be traced back over 3000 years. Qigong exercises have been prescribed by Chinese doctors much the same way Western doctors prescribe physical therapy. Qigong is considered as a self-training method of regulating body posture, breathing, and mind, with the overall aim being to achieve an optimal state of body and mind [13]. Hallmark features of qigong movements and forms include patterns of slow, rhythmic movements where attention is paid to posture, breathing and inner balance. This enhances the functioning of the autonomic nervous system and strengthens the overall state of health [14,15]. Physical benefits of the practice of qigong include improved balance, relaxation, concentration, and coordination [16,17,18]. Qigong is accessible to people of all ages, abilities, and physical strength. It is low cost and easy to learn and has few known side effects.

## 2. Purpose

Existing research provides little detail on the mechanisms of qigong intervention and individual response to treatment. As treatment and follow-up is most often a short-term outcomes assessment, a study of long-term Qigong practice and follow-up assessment was explored. The following study fully describes the qigong intervention as a standardized form of treatment and allows the subjects to describe the effects of their Qigong practice (24-Posture Qigong) on manifestations of LBP pathology and related symptoms.

## 3. Methods

### 3.1. Design and Subjects

This study is designed as a descriptive discussion of two case studies. The subjects each had significant history of medically confirmed chronic LBP with functional loss. The subjects were ones of convenience, as was the qigong form practiced. The subjects were selected by their teaching master, who approached them to identify their willingness to interview with the intent to publish their case study stories.

Data were obtained retrospectively through direct interviews with the two subjects. The first author, a doctor of physical therapy with orthopedic experience and an instructor in the 24-Posture exercise regimen, conducted the interviews. He had no prior relationship with the subjects.

Subjects were informed by the interviewer as to the purpose of the interviews with respect to intent to publish. Procedures were limited to interview so no physical risks were present. Subjects were informed of their right to withdraw at any time during the interview. The final report was shared with each subject to review it for accuracy and final approval for publication. Names have been changed in the reporting to keep identities confidential.

Both subjects have practiced this exercise regimen for approximately 4–5 years; the following report discusses relevant medical diagnoses (LBP in particular) and past medical management, their experiences with the 24-Posture Qigong and its effects on their symptoms, functional activities, and quality of life. These constructs were organized as categories and presented in a comparative table, and as a descriptive discussion.

### 3.2. Intervention

Both subjects in this case report practice the modern 24-Posture Therapeutic Qigong as taught by a fourth-generation master in the *Wu Yi Jie He* Family System of Tai Chi and Qigong. The exercises are part of the *Wu Yi Jie He* Family System which originally included 20 postures designed by Dr. (Grandmaster) Wang Zi Ping, and systematized by Dr. Wu Chengde. *Twenty Therapeutic Exercises for Treating Diseases and Prolonging Life* was published in 1958. The Family System is based on ancient qigong exercises, but also incorporates Dr. Wang Zi Ping’s decades of clinical experience. Its origins can be traced back centuries. In its current form, the qigong exercise regimen includes 24 dynamic postures, each performed slowly and rhythmically with breath control and mental intent. Four new postures were added in a revision by Grand Master Helen Wu, granddaughter of Dr. Wang Zi Ping. This modern form has been used for more than four decades. The design of the series of 24 postures follows the human spine and the command system of the body—the nervous system—through the neck, then the back, and through the limbs [19]. Please refer to Table 1 for comparison of subjects.

## 4. Results

### 4.1. Case Descriptions

#### 4.1.1. Susan

Susan is a 51-year-old woman. As an adolescent, she developed scoliosis. Working as a hairstylist when she was 18 or 19, she began to have severe pain in her hands, making her work very difficult. She had carpal tunnel surgery on her right hand which provided temporary relief. Her symptoms slowly returned and she continued to have intense pain and difficulty with her daily activities. Seeking relief from her symptoms, she received treatment from chiropractors and Chinese medicine practitioners with no significant benefit. She also tried yoga and swimming but depending on the yoga movements or swimming strokes, she began to have more pain.

When she was in her early forties, she began to have weakness in the lower half of her body. Her legs would frequently give out on the stairs, causing her to fall. Control of her bladder and bowel function also began to deteriorate. She had an MRI, and based on the results, she was scheduled for emergency surgery to stabilize her cervical spine and relieve spinal cord compression. She was informed that it would be an outpatient surgery and she would be home the same day.

In May 2011, Susan had a 6-level cervical spine fusion to stabilize her spine and relieve pressure on her spinal cord. When she woke up, she realized something was wrong. The entire right side of her body was paralyzed. What was supposed to be an outpatient surgery with a return home the same day ended up being an 8-week hospital and rehabilitation stay to learn how to walk again.

In August 2012, a second surgery was performed with the goal of pain relief. However, she reported her pain was significantly worse afterwards. She would receive weekly injections for pain relief that she reports would only marginally decrease her pain for about one week. She was also taking high doses of medication for nerve pain relief which she also reports provided marginal benefit. She had areas of heightened sensitivity on her legs where a bed sheet or even a gentle breeze would cause intense pain. Exposure to hot water would feel cold, and cold water would feel hot. She was unable to walk more than one block and remained in bed for over ten hours per day. She suffered extensive personal and professional quality of life losses at this time.

Looking for relief, she tried medical yoga and received temporary relief. She also practiced traditional Tai Chi which provided minimal to no relief. She continued to have intense pain which had a significant impact on her quality of life and ability to perform daily tasks, including walking. In 2014, she tried a Qigong class and reported feeling that there was something very different about this class. Almost immediately, she felt a strong sense of relaxation that she had reportedly not found in many years. She was unable to attend many classes, so she purchased a video of the movements. Over the next year, she began to practice the exercises consistently until she could attend formal classes.

Susan credits the practice of qigong with saving her life. She says it gave her a reason to get out of bed and socialize in the very early days of her practice. She is now able to walk with no limitations and her pain has improved by approximately 90%. Within three months of beginning qigong, she was able to stop all medications and injections she was receiving for pain relief. She continues to have some weakness in her arms and her hands, but it does not interfere with her ability to complete her daily activities. Despite her extensive cervical spine surgery, she reports full mobility in her shoulders and has nearly recovered full mobility in her neck. Her energy has also greatly improved and she routinely teaches three Qigong classes per week. In addition to this, she teaches at special events, including a Qigong class at a large yoga festival with over 1000 people in attendance. Recently, she started her first full time job in over six years.

#### 4.1.2. James

James is a 70-year-old male. Over ten years ago, he was diagnosed with multi-level degenerative disc disease (DDD) in his lumbar spine, as well as severe central stenosis or narrowing of his spinal column around his spinal cord at L3-4, L4-5, and L5-S1. In 2011, he had a CT scan and was told by his physician that it would not be long until he must rely on a wheelchair for all mobility. Surgery was presented as an option, but he was informed that the success rate was less than 10%.

He decided to forgo surgery and take his chances. Gradually, his legs became weaker and he would fall spontaneously. He worked as a salesman and as he was talking to clients, his legs would give out without warning and he would fall to the ground. To help his situation, he would park as close as he could to the entrance of stores or other destinations and would walk with carts or holding onto shelves or furniture. This would only help for so long before he would fall again.

He began looking for other options to help manage his condition. In 2012, he tried yoga. While it helped to temporarily control his pain, it had no effect on the weakness in his legs and he would continue to fall. One year later, at the suggestion of his wife, he tried a Qigong class. Due to his family’s personal schedules, he was unable to attend another class for two months. He decided to purchase a video of the movements he had learned and practiced them each day for 4–5 months until he was able to return to normal classes.

Since participating in Qigong classes, he has not fallen even once, and has no reports of pain. He stated that “Qigong gave me my life back.” He has no other medical problems to mention and takes no medications. He revealed that the improvements he has experienced have gone far beyond what he expected. Where he used to fall often and without notice, he has not fallen since he began qigong and reports that he now even has a “spring to his step, and a spring in his heart.” Not too long ago, he sustained a left rotator cuff tear. After continuing to practice qigong, he had a full return of strength and movement with no pain or difficulty with his routine daily activities, all within six months.

In his professional life, he felt like he was burning out as an IT programmer but practicing qigong has reinvigorated him. He was able to complete many projects (some complex) that he never would have thought possible. He has since become certified to teach Qigong and tells anyone who will listen about his story. He has also witnessed many others gain significant benefit from the practice of Qigong and is thrilled that he gets to share this with others.

## 5. Discussion

Studies of the effectiveness of qigong for LBP are limited. Blodt and colleagues compared Qigong and an exercise program consisting of general strengthening and stretching exercises and their effects on chronic LBP. Patients were randomly assigned to a qigong group (*n* = 64) which practiced 1× weekly for 90 minutes over three months, or an exercise group (*n* = 63) which also practiced 1× weekly for 60 minutes over three months. No significant differences between qigong and exercise were found in pain intensity, disability, quality of life, sleep quality or satisfaction with sleep after a 3, 6, and 12 month follow up period [20]. A recent systematic review and meta-analysis by Zou and colleagues concluded that Baduanjin, a type of qigong exercise, has the potential to reduce musculoskeletal pain and improve overall sleep quality in people with chronic illness [21]. Illnesses in the study included insomnia, body pain (shoulder, neck, and/or back), periarthritis, ankylosing spondylitis, lumbar disc herniation, osteoporosis, type 2 diabetes mellitus, radiculopathy, Parkinson’s disease, chronic fatigue syndrome-like illness, and hypertension.

Qigong has also been shown to be effective in treating neck pain. Patients aged 40–60 years with chronic neck pain of approximately 3.2 years (±1.6 years) were randomly assigned to qigong (*n* = 42), exercise (*n* = 39), or wait list controls (*n* = 41). After a six month intervention, both Qigong and exercise therapy participants gained significant improvement in neck pain, disability and quality of life over those who received no treatment [22].

Tai Chi, which is based on the same principles and practices as Qigong, has also demonstrated positive effects in regard to LBP. In addition, many studies reporting on what is described as tai chi are modified to make the practices shorter and easier to learn, thus making them very similar to qigong movements [23]. Due to the similarities between tai chi and qigong, and the realization that tai chi protocols are often adapted to resemble qigong practice, it allows us to suggest that outcomes can be counted across both types of studies (further supporting claims of equivalence) [23].

In a randomized controlled trial of 160 volunteers aged 18 to 70 years with constant non-specific LBP, tai chi exercise reduced how often back symptoms interfered with daily activities. It also resulted in reduced pain intensity and improved self-reported disability on the Roland-Morris Disability Questionnaire scale compared to the control group (which continued with routine care). Results showed that a 10-week tai chi program improved pain and disability outcomes and can be considered a safe and effective intervention for those experiencing constant, long-term LBP symptoms [24]. A systematic review and meta-analysis of 18 randomized controlled trials on tai chi has also shown favorable results, demonstrating immediate relief of chronic pain from osteoarthritis. The recommended duration of Tai Chi practice to receive benefit from osteoarthritis symptoms may be more than five weeks. These studies also provide evidence regarding the beneficial effects of Tai Chi on the immediate relief of chronic pain due to LBP and osteoporosis [25].

Results of the current report reveal that qigong may also provide individuals with relief from chronic LBP. Both subjects in this report had chronic LBP for which they sought traditional medical care with limited benefit. Both subjects sought holistic forms of treatment, including qigong. As qigong is a form of self-stimulation of acupuncture points using the same energy theory as passive acupuncture, qigong practice has added value of self-efficacy compared to passive alternative treatments such as acupuncture. Master instructors purport that daily practice of the 24-Posture regimen can reduce stress and tension, and regulate and promote homeostasis resulting in healing, health and longevity. These claims are supported by decades of clinical observation, but as yet no controlled clinical trial has been conducted to validate this theory. As a first step in the exploration of the therapeutic benefits of a standardized program for comprehensive Qigong exercise practiced for 20–40 min daily, this paper offers a qualitative presentation of two case studies by providing some insight into mechanisms of treatment and individual response.

Both subjects initially attempted the practice on the suggestion of others. Years later with no additional medical care, they both report that they have significantly improved their conditions. For Susan, her pain has improved by at least 90% and no longer interferes with her daily activities. For James, he no longer has pain or weakness in his legs that would cause him to fall at any time without warning. Both subjects report a return to all previous activities with more energy and vigor. Throughout their experience, they reported no evidence of negative side effects as a result of Qigong practice. Qigong can be offered at minimal cost when compared to traditional medical care or alternative acupuncture. Further, Susan and James had the convenience of practicing whenever they wanted to, at their own pace.

Common themes reported by both subjects are self-referral resulting from conversations with family or friends in the community, and that they both reported a sense of empowerment after beginning to practice Qigong. They both report more control over their health and bodies than they had previously thought, rather than relying on someone else to “fix them”.

The questions of relevance regarding these two case studies are: what mechanisms of postural imbalance might cause dysfunction, and what mechanisms in the practice of the 24-Posture Qigong regimen might have influenced outcomes? Theoretically, chronic pain as experienced by these two individuals may result from long-term sensitization and loss of function due to incoming nociceptive signals (i.e. the intermittent loss of nerve control to anti-gravity muscles in the legs as experienced by both subjects in this report). The practice of mindful movement is known to induce a relaxation effect and systematic regulation of inflammation. The gentle mechanical articular stimulation of the flowing qigong movements has the potential to reduce inflammation at a local level, reduce postural imbalance due to muscle guarding, and to promote tissue repair. Postural correction optimizes the position of the diaphragm, resulting in increased oxygen levels and a gentle mechanical massage to the viscera. Postural correction of the bony spine has the potential to optimally orient the pelvic bowl and retrain core stabilizing muscles. Finally, relief of symptoms and postural correction may have a positive emotional somatic response.

## 6. Future Research

These two case studies give rise to questions for further study. Foremost among them are mechanistic theory validation and prospective, controlled, long-term study. Recommended objective measures based on subjects’ report of benefits include pain, physical function, sense of well-being, postural assessment, and self-efficacy.

Given that large-scale, long-term clinical research may take a decade or more to guide prescriptive treatment with Qigong (if performed at all), the current advice for Qigong clinicians when approached by individuals with similar complaints as Susan or James is to first recognize neural involvement and request medical clearance before beginning a course of Qigong instruction and practice. Once medically cleared for gentle and mindful exercise, individual experience may be the most relevant measure of intervention success.

## 7. Ethics in Practice

Methods were approved by a sanctioned Institutional Review Board (IRB). Study methods presented no physical or psychological risk to the two subjects. No deception was employed. Both subjects were fully informed of the purpose and intended disposition of the study.

## 8. Conclusions

Long-term daily practice of a standardized regimen of qigong exercise was found to have a clinically significant reduction in symptoms and restore physical function in two individuals with chronic back pain. Results were attributed to postural correction, release and correction of muscle imbalance, pain management, and restoration and re-education of neuro-control. It is recommended that future research address theory validation through prospective, controlled, long-term study.

## Figures and Tables

**Table 1:** Comparative description of two cases studies.

	Susan	James
**Age/Sex**	51-year-old female.	70-year-old male.
**Diagnosis**	Spinal cord compression, status post spinal fusion with constant widespread pain.	Multi-level lumbar degenerative disc disease with central and foraminal stenosis.
**Prior Interventions**	Multi-level cervical spine fusion with subsequent revision; physical therapy; pain injections and medications, chiropractic; yoga.	Medical assessment undertaken but chose not to follow a recommendation for surgical intervention. Tried yoga with little or no relief.
**Symptoms prior to Qigong**	Severe pain throughout her body; severe fatigue; poorly controlled hypertension (HTN), type II diabetes (DM).	Weakness, frequent sudden falls (several per week).
**Qigong Practice**	4 years Often home practice with aid of an instructional DVD. Currently attending classes and regularly leads qigong classes in the community.	5 years Often home practice with aid of an instructional DVD. Currently attending classes as his time permits and continues to diligently practice at home.
**Self-reported outcomes**	90% improvement in pain; improved endurance and mood states; hypertension, diabetes mellitus, and cholesterol well controlled; able to obtain full-time employment.	No falls since beginning qigong; improved energy and motivation to pursue his life’s work.
